# Reversible Thermo-Responsive Valve for Microfluidic Paper-Based Analytical Devices

**DOI:** 10.3390/mi13050690

**Published:** 2022-04-28

**Authors:** Hiroki Toda, Wataru Iwasaki, Nobutomo Morita, Taisei Motomura, Kenshin Takemura, Masaya Nagano, Yoshitaka Nakanishi, Yuta Nakashima

**Affiliations:** 1 Graduate School of Science and Technology, Kumamoto University, Kumamoto 860-8555, Japan; 209d3123@st.kumamoto-u.ac.jp (H.T.); 181t2209@st.kumamoto-u.ac.jp (M.N.); 2 Sensing System Research Center, National Institute of Advanced Industrial Science and Technology, Saga 841-0052, Japan; morita.nobutomo@aist.go.jp (N.M.); t.motomura@aist.go.jp (T.M.); takemura.kenshin@aist.go.jp (K.T.); 3 Faculty of Advanced Science and Technology, Kumamoto University, Kumamoto 860-8555, Japan; y-naka@mech.kumamoto-u.ac.jp; 4 Institute of Industrial Nanomaterials, Kumamoto University, Kumamoto 860-8555, Japan; 5 International Research Organization for Advanced Science & Technology, Kumamoto University, Kumamoto 860-8555, Japan

**Keywords:** microfluidic paper-based analytical devices, thermo-responsive polymer, poly(*N*-isopropylacrylamide), valve, plasma-induced graft polymerization

## Abstract

Fluid control on a paper channel is necessary for analysis with multiple reagents, such as enzyme-linked immunosorbent assay (ELISA) in microfluidic paper-based analytical devices (µPADs). In this study, a thermo-responsive valve was fabricated by polymerizing N-isopropylacrylamide on a PVDF porous membrane by plasma-induced graft polymerization. The polymerized membrane was observed by scanning electron microscopy (SEM), and it was confirmed that more pores were closed at temperatures below 32 °C and more pores were opened at temperatures above 32 °C. Valve permeability tests confirmed that the proposed polymerized membrane was impermeable to water and proteins at temperatures below 32 °C and permeable to water at temperatures above 32 °C. The valve could also be reversibly and repeatedly opened and closed by changing the temperature near 32 °C. These results suggest that plasma-induced graft polymerization may be used to produce thermo-responsive valves that can be opened and closed without subsequent loss of performance. These results indicate that the thermo-responsive valve fabricated by plasma-induced graft polymerization could potentially be applied to ELISA with µPADs.

## 1. Introduction

**Figure 1:**
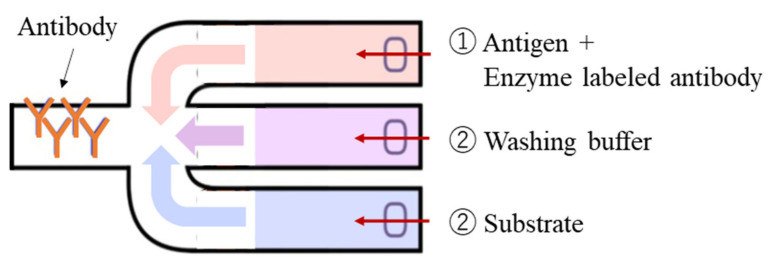
Schematic of an enzyme-linked immunosorbent assay (ELISA) on a μPAD.

Microfluidic paper-based analytical devices (µPADs), which use paper as a flow path [1,2,3,4], present the advantages of abundance, low cost, a low volume of reagent used, easy disposal, ease of operation, environmental friendliness, and compatibility with biological samples. A major feature of µPADs is that they do not require a pump for reagents and samples because they flow due to the capillary force of the paper substrate, resulting in a compact system. Because of these features, μPADs are expected to be applied to point-of-care testing (POCT), which is used in various locations and situations that require immediate results such as testing for infectious diseases [5]. POCT is a system of testing that provides patient examination and immediate results that may be used in medical care. Immunochromatography, a technique similar to µPADs, is widely used for POCT. Immunochromatography is an immunoassay method that utilizes the slow capillary flow of a test sample across a nitrocellulose (NC) membrane while dissolving the reagent. The antigen in the specimen moves across the cellulose membrane, forming an immunocomplex with the labeled antibody prepared in advance, and the antigen is trapped on the capture antibody prepared on the NC membrane, which is then visually detected. Immunochromatography has been applied to the diagnosis of various conditions such as pregnancy and influenza. While advantages of this method are that it does not require any analysis equipment and is easy to use because it provides a visual diagnosis, it does not allow for quantitative measurement and can lead to individual errors in judgment. These disadvantages may be resolved by performing an enzyme-linked immunosorbent assay (ELISA) with μPADs [6,7,8]. ELISA is the most common type of immunoassay, in which a sample, an enzyme-labeled antibody, and a substrate are sequentially introduced into a reaction field (usually a 96-well plate) on which the antibody is immobilized, interspersed with washing operations, and the concentration of the antigen is measured using the reaction volume of the enzyme and substrate. To perform analysis using multiple reagents such as ELISA on μPADs, it is necessary to set the reagents in multiple channels and allow them to flow in sequence (Figure 1).

**Figure 2:**
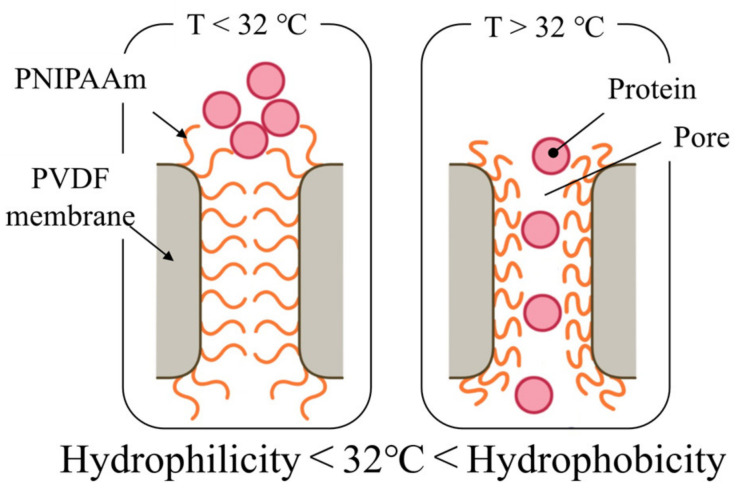
Mechanism of a thermo-responsive valve on a μPAD for performing ELISA.

Due to the difficulty of fluid control on paper, analysis accuracy is reduced because of backflow and mixing of reagents in the flow channel. Therefore, various flow control methods have been developed for μPADs [9,10,11,12,13,14,15,16,17,18,19,20]. Hao Fu et al., used polyolefin and thermo-responsive shape-memory polymer (SMP) as a valve by thermally controlling the angle of the cantilever arm with a paper bridge, and successfully automated ELISA on a μPAD [9]. Fragments of the SMP may be deformed from a permanent shape to a temporary shape when heated to a temperature above the switching transition temperature (T_trans_) and maintain the temporary shape when cooled to a temperature below T_trans_. When heated again at a temperature above T_trans_, the memory shape polymer returns to its permanent shape. Using this property, a thermo-responsive memory shape polymer was introduced into the device design to enable bi-directional actuation of the paper valve and automatic control of the fluid on the μPAD to perform ELISA. However, the T_trans_ of polyolefin is 95 °C, which implies that the valve must be heated above 95 °C each time it is turned on or off. Raising the temperature from room temperature to 95 °C requires time, resulting in low valve responsiveness and high energy requirements. To shorten the analysis time compared to that of SMP valves, the valve must be capable of switching and must have a high responsiveness. Therefore, we previously developed a valve mechanism using a thermo-responsive polymer as a fluid control technology that was applicable to μPAD. A hydrophilic PVDF porous membrane was used as the base material of the valve, and poly(N-isopropylacrylamide) (PNIPAAm) was used as the thermo-responsive polymer. PNIPAAm exhibits hydrophobicity changes at 32 °C [21,22]. Therefore, we may open and close the pores by controlling the PNIPAAm temperature at over and under 32 °C due to swelling and dehydration (Figure 2). We previously achieved polymerization under various conditions to find the appropriate polymerization conditions for this system. However, although it is expected that different amounts of polymerization would affect flow velocity after the reagent passes through the valve, the relationship between amount of polymerization and flow velocity has not yet been evaluated [23]. In this study, the relationship between the amount of polymerization and flow rate, the permeability of the protein for ELISA, and the reversibility of valve opening and closing were investigated.

## 2. Materials and Methods

### 2.1. Material

For this study, we purchased polyvinylidene fluoride (PVDF) membranes (Durapore^®^ membrane filter, HVLP02500), NC membranes (Hi-Flow™ Plus HF120), and absorbent pads (SureWick^®^) from Merck Millipore Ltd. (County Cork, Ireland). We purchased NIPAAm, *N*,*N*′-methylenebisacrylamide (BIS), and ammonium peroxodisulfate (APS) from Sigma-Aldrich Japan (Tokyo, Japan), and polydimethylsiloxane (PDMS, SILPOT 184) from Dow Corning Toray Co., Ltd. (Tokyo, Japan). Alkaline phosphatase (ALP) labeled antibody was purchased from Acris Antibodies (AM08098AP-N, San Diego, CA, USA) and 5-bromo-4-chloro-3-indolyl-phosphate/nitroblue tetrazolium (BCIP/NBT) tablets from Sigma-Aldrich Japan (B5655, Tokyo, Japan). We used ultrapure water with a conductivity of 18.2 MΩ·cm in all experiments.

### 2.2. NIPAAm Polymerization on a PVDF Porous Membrane

**Figure 3:**
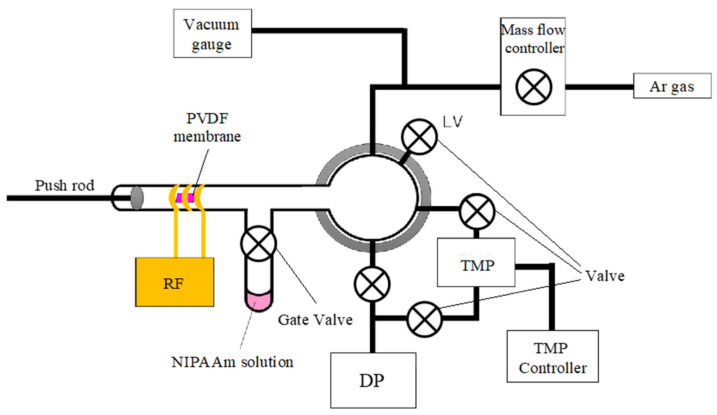
Schematic of the proposed plasma-induced graft polymerization system. A PVDF membrane can be dropped into the NIPAAm solution without exposure to the atmosphere by pushing with a push rod after plasma irradiation. RF; radio frequency power source. TMP; turbo molecular pump. DP; diaphragm pump.

A plasma-induced graft polymerization method with argon plasma was used to polymerize NIPAAm on a PVDF porous membrane. When a PVDF porous membrane is irradiated with plasma, radicals are generated. The PVDF porous membrane used in this study was polymerized by immersion in NIPAAm solution and incubation at 30 °C for 5 h. Because the polymerization reaction was inhibited by oxygen, it was necessary to immerse the PVDF in NIPAAm solution after plasma irradiation without exposing it to the atmosphere. We used a previously developed plasma-induced graft polymerization system that may be used to perform this series of processes (Figure 3). The NIPAAm monomer that was purified by recrystallization from hexane was dissolved in 50/50 vol% methanol/water solution. BIS and APS were dissolved in the NIPAAm solution to provide molar ratios of 0.5 and 1% for the NIPAAm monomer, respectively. The concentration of NIPPAm monomer was varied between 2.0 wt% to 3.0 wt%. The NIPAAm solution was poured into a glass bell jar. The PVDF membrane was placed in the glass nipple so that it was in the center of the coil wire. The glass nipple and glass bell jar containing the PVDF membrane and NIPAAm solution were roughly evacuated with a diaphragm pump (DP) to degas the NIPAAm solution. Next, the gate valve connecting the glass nipple and glass bell jar was closed, and the glass nipple was evacuated using a turbo molecular pump (TMP). Once the vacuum reached a value below 10^−5^ Torr, argon gas was introduced into the glass nipple at a flow rate of 30 sccm, and the PVDF membrane was subjected to 20 W inductively coupled plasma for 30 s. After all the argon gas was introduced and the vacuum exhaust systems were stopped, the gate valve was opened and the PVDF membrane was pushed using a push rod and dropped into the NIPAAm solution. The gate valve was then closed again, and the gate valve and glass bell jar containing the NIPAAm solution and PVDF membrane were removed from the apparatus and incubated at 30 °C for 5 h to initiate polymerization of NIPAAm. After incubation, the PVDF-PNIPAAm membrane was immersed in ultrapure water and shaken for more than 12 h for cleaning.

### 2.3. Membrane Characterization Method

#### 2.3.1. Scanning Electron Microscopy of Membranes

Changes to the surface morphology of the fabricated PVDF-PNIPAAm membranes with a changing temperature were observed using scanning electron microscopy (SEM). To observe the changes in the surface morphology even in a vacuum, samples for SEM observation were prepared according to the method described in the previous work {Xiao, 2014 #219}. Two pieces were cut from the same PVDF-PNIPAAm membrane and immersed overnight in ultrapure water at 25 °C and 40 °C, which is far away from the lower critical solution temperature (LCST) of NIPAAm (32 °C). The specimens were then instantly frozen in liquid nitrogen. The membranes were freeze-dried in a freeze-dryer (FD-1000; Tokyo RIKAKIKAI Co. Ltd., Tokyo, Japan) and gold was deposited on the surface using a sputtering apparatus (SC-701HMCII; Sanyu Electron Co. Ltd., Tokyo, Japan) and observed using SEM (JSM-6701F, JEOL Ltd., Tokyo, Japan). In this study, only the surface of the PVDF-PNIPAAm membrane was observed because it was difficult to observe the membrane interior.

#### 2.3.2. Valve Performance Evaluation

**Figure 4:**
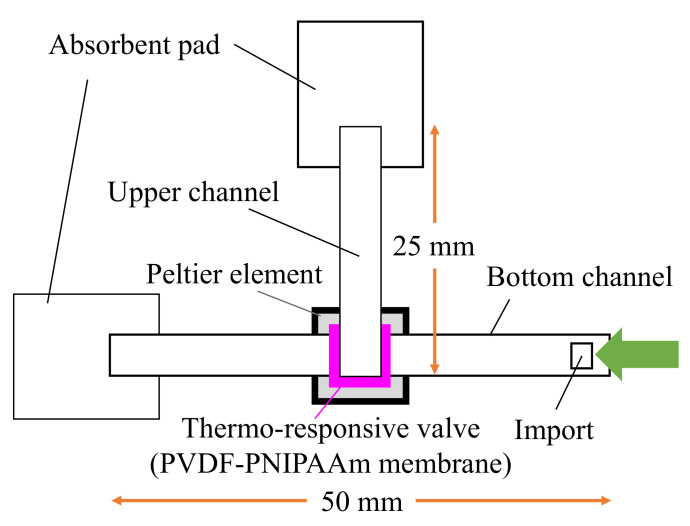
Schematic of the evaluation system used to assess valve performance. The polymerized membrane was stacked in a T-shape between two nitrocellulose membranes, and colored water flowed through the import. If the valve was closed, the water flowed only in the horizontal direction; if the valve was open, it flowed in the upper channel. The temperature of the valve was controlled by adjusting the current flowing through the Peltier heater.

To evaluate the efficacy of the fabricated PVDF-PNIPAAm membrane as a valve, we used a system of valve performance testing (Figure 4). NC membranes, which are paper flow channels, were stacked in a T-shape with a valve, and green colored water was injected at one side of the bottom channel at 25 °C. The green dye flowed through the bottom NC membrane towards the other edge. At 240 s after injection, the valve was heated to 40 °C, and flow behavior was observed. When the valve was closed, the colored water only flowed through the bottom channel; when the valve was opened, it flowed also through the upper channel. Using this system, we confirmed that the fabricated PVDF-PNIPAAm membranes functioned as valves.

## 3. Results and Discussion

### 3.1. Polymerization of the Proposed Membrane

**Figure 5:**
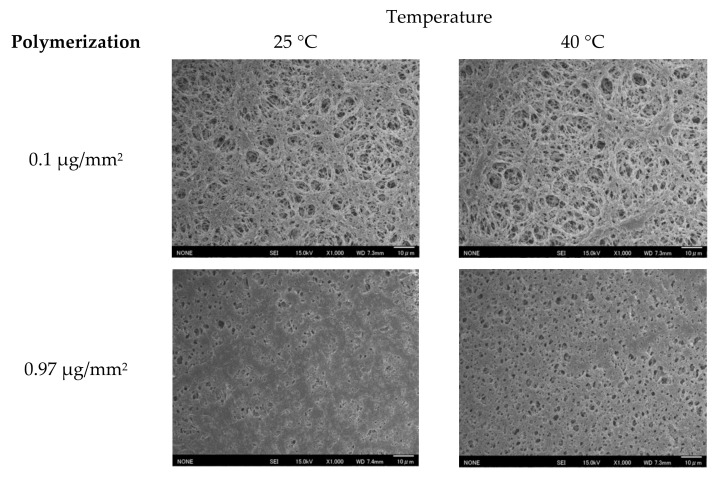

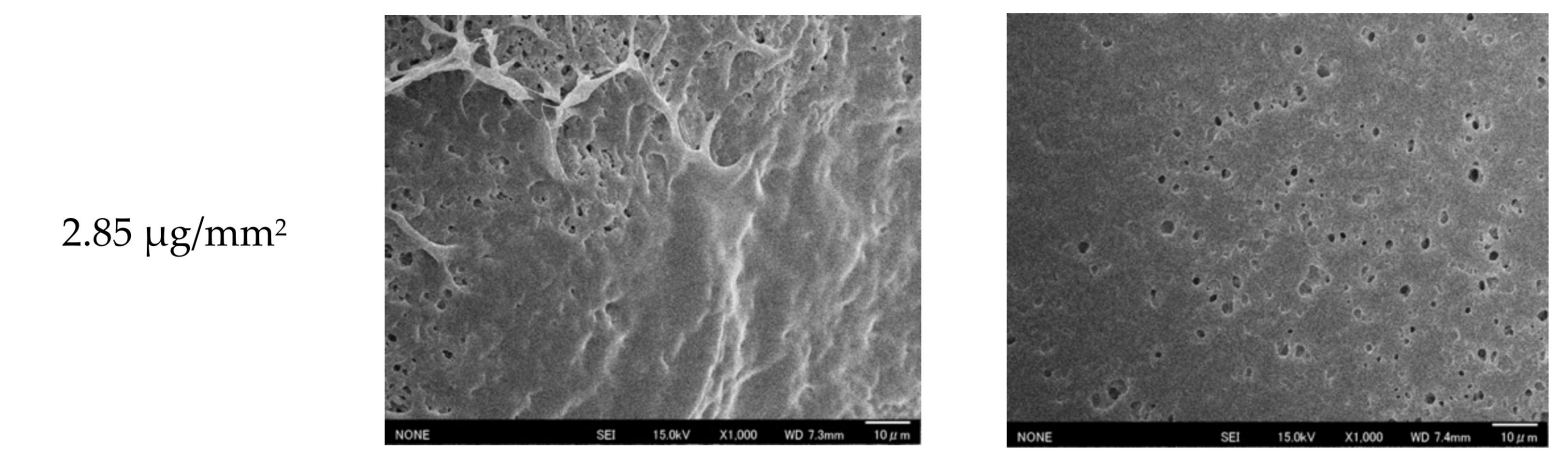
SEM images of PVDF-PNIPAAm membranes with different amounts of polymerization at 25 and 40 °C.

PNIPAAm was polymerized on PVDF porous membranes by plasma-induced graft polymerization. The difference between the membrane mass before and after polymerization was measured as an amount of polymerization. The SEM images of membranes with 0.1, 0.97, and 2.85 μg/mm^2^ of polymerization are shown in Figure 5. No significant differences in pore opening were observed between 25 and 40 °C for a PVDF-PNIPAAm membrane with 0.1 μg/mm^2^ of polymerization. However, PVDF-PNIPAAm membranes with 0.97 and 2.85 μg/mm^2^ of polymerization showed more pores and larger pore sizes at 40 °C than at 25 °C. The membrane with 2.85 μg/mm^2^ of polymerization had fewer pores at both temperatures compared to 0.97 μg/mm^2^.

### 3.2. Valve Function of Thermo-Responsive Valve Membrane

**Figure 6:**
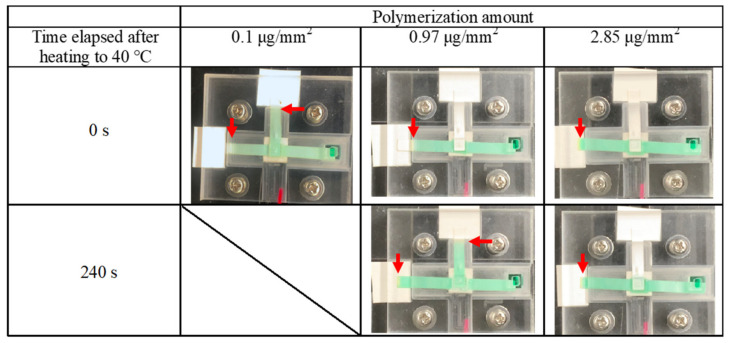
Time-lapse photographs of colored water flowing into the valve function evaluation system, in which each of the membranes with different amounts of polymerization were installed. Colored water was flowed at 25 °C for 240 s before heating of the valve. The valve was heated to 40 °C. Arrows indicate the position of the wetting front.

The valve performances of PVDF-PNIPAAm membranes were evaluated. The results of performance tests on PVDF-PNIPAAm membranes with 0.1, 0.97, and 2.85 μg/mm^2^ of polymerization are shown in Figure 6. In the performance test of the PVDF-PNIPAAm membrane with 0.1 μg/mm^2^ of polymerization (Video S1), the valve opened and flow was observed into the upper channel, even at 25 °C. Performance tests on PVDF-PNIPAAm membranes with 0.97 μg/mm^2^ of polymerization showed that liquid flowed only in the bottom channel at 25 °C, and the valve opened and liquid flowed into the upper channel at 40 °C (Video S2). In the case of the PVDF-PNIPAAm membrane with 2.85 μg/mm^2^ polymerization (Video S3), the valve closed at 25 °C and liquid flowed only into the bottom channel. Moreover, at 40 °C, the valve opened, but little liquid flowed into the upper channel. SEM observations and the results of performance tests show that at 25 °C, if both the membrane surface pore size and number of pores are low, the membrane prevents liquid from permeating through it. At 40 °C, the rate of permeation through the membrane was found to vary depending on pore diameter and number of pores.

**Figure 7:**
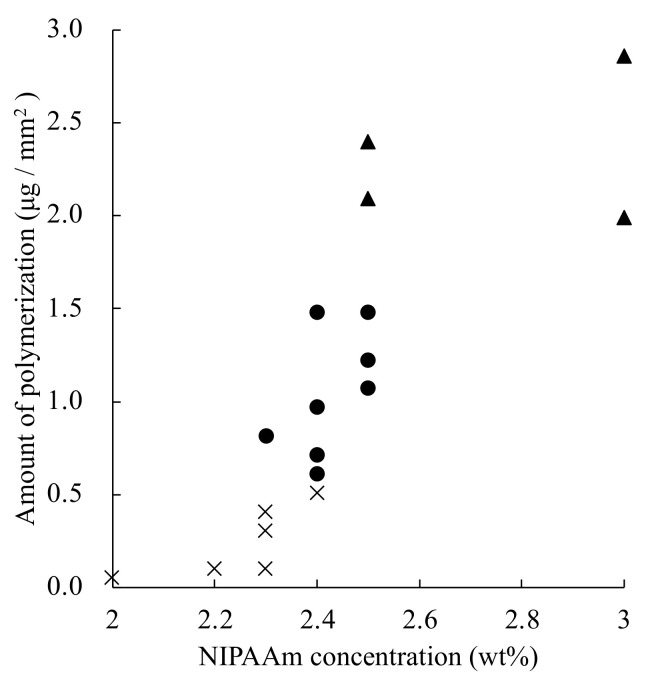
Relationship between polymerization of PNIPAAM and valve performance. ×: the valve did not close, even at 25 °C, ●: the valve showed good valving performance, and ▲: poor permeability was observed after valve opening.

Relationship between NIPAAm concentration and the amount of polymerization and the results of performance tests with other amounts of polymerization in the membranes are shown in Figure 7. A tendency for the amount of polymerization to increase with higher NIPAAm concentrations was observed. It may be proposed that the amount of polymerization varied even when the concentration of NIPAAm was constant because NIPAAm polymerization continued to progress during the vacuuming process, and its polymerization rate was affected by environmental temperature. The PVDF-PNIPAAm membrane with the polymerization amount between 0.61 and 1.48 μg/mm^2^ operated properly as a valve. It was considered that pores on the surface of the PVDF-PNIPAAm membrane opened and closed normally at polymerizations between 0.61 and 1.48 μg/mm^2^. For polymerizations lower than 0.61 μg/mm^2^, the pores did not close, and for polymerizations higher than 1.48 μg/mm^2^, the pores were prevented from opening. From this result, we defined the PVDF-PNIPAAm membrane with the polymerization amount to be between 0.61 and 1.48 μg/mm^2^ as a good valve membrane.

**Figure 8:**
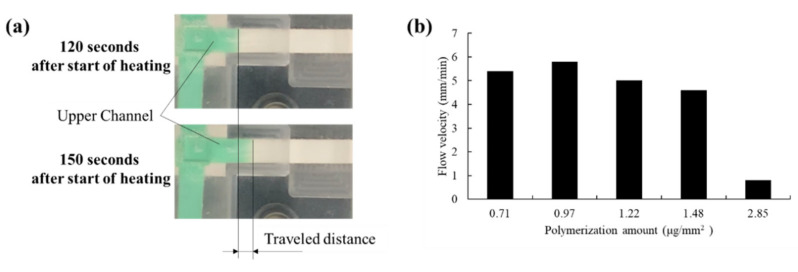
Comparison of flow velocities in the upper channel for membranes with various amounts of polymerization. (**a**) Method for measuring flow velocity. (**b**) Relationship between flow velocity and amount of polymerization. Flow velocity was calculated by measuring the difference of the distance of the wetting front at 120 and 150 s after heating was started.

The permeability rates of valves with 0.71, 0.97, 1.22, 1.48, and 2.85 μg/mm^2^ of polymerization were evaluated (Figure 8). Flow velocity was calculated by measuring the difference of the distance of the wetting front at 120 and 150 s after heating was started. At a polymerization of 0.71~1.48 μg/mm^2^, the flow velocity tended to decrease with increasing polymerization volume. However, considering that the nominal value for the variation in capillary flow time specification of the nitrocellulose membrane used is 120 ± 30 s/4 cm, these differences are not expected to have a significant impact. The membrane with a polymerization of 2.85 μg/mm^2^ presented a low flow velocity of only 0.8 mm/min. This may be due to the fact that the number of pores was low even at 40 °C as shown in Figure 5.

### 3.3. Protein Permeability

**Figure 9:**
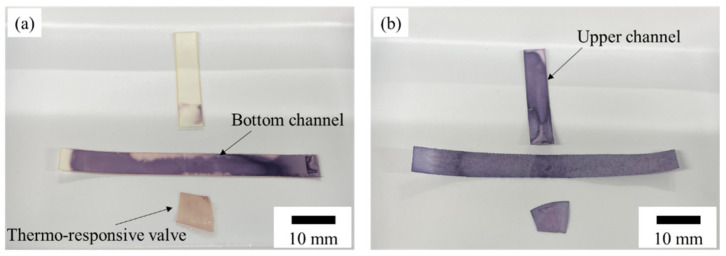
Flow channel and valve immersed in BCIP/NBT solution after ALP flowed through the valve performance evaluation system. (**a**) ALP flowed without heating the valve, and (**b**) ALP flowed in the case of valve heating.

When performing ELISA with µPADs, it is necessary to use antibodies and enzymes such as horseradish peroxidase (HRP) and ALP. Their size is about 5~15 nm [24,25,26], which is sufficiently smaller than the several µm pore diameter confirmed from the SEM image in Figure 5, but since there is a possibility of protein adsorption, protein permeability was verified. In this experiment, an ALP-labeled antibody, which was expected to be used in ELISA with µPADs, and a valve with the polymerization amount of 1.48 µg/mm^2^ were used. We performed the experiment in two conditions, where the valve was unheated or heated to 40 °C from the beginning. The ALP-labeled antibody diluted 500-fold in pH 7.4 Tris-buffered saline containing 0.05% Tween20 and 0.1% bovine serum albumin was injected into the evaluation system, the experiment was ended after 8 min, and the flow channel was disassembled. The two NC membranes and the valve removed from the disassembled evaluation system were immersed in BCIP/NBT solution, a colored substrate that reacts with ALP, and the permeability of ALP was evaluated (Figure 9). The experiment was performed twice, once without heating the valve (a) and then with valve heating (b). In case (a), the upper channel was not colored, while in case (b), the upper channel was colored. These results indicate that PVDF-PNIPAAm membranes can control flow even in the case of input proteins, which suggests that they are applicable to ELISA.

### 3.4. Valve Reversibility

**Figure 10:**
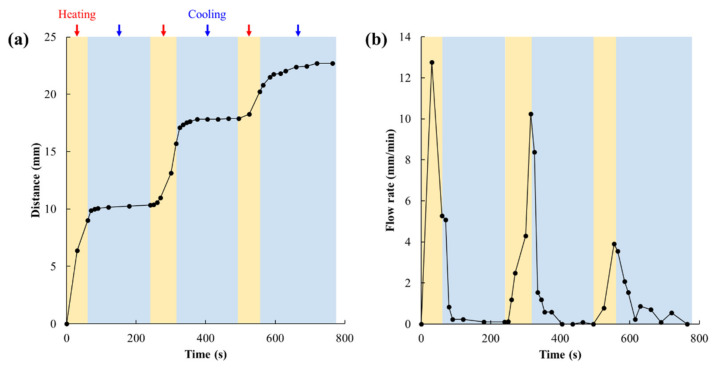
Flow behavior when the proposed valve is repeatedly opened and closed. (**a**) Distance of the wetting front of the green colored water in the upper channel and (**b**) its flow velocity. The distance traveled by the wetting front of the green colored water in the upper channel was measured every 10 to 30 s (**a**), and flow velocity was calculated from the difference between the distance traveled at a point and distance traveled at the previous measurement point (**b**). Orange-shaded areas indicate heating above 32 °C, and blue-shaded areas indicate cooling below 32 °C.

Closing the proposed valve after the minimum necessary amount of reagent has flowed would imply quicker drying of the analyzer. As the analyzer dries, the reagent concentrates, and the reaction rate increases. Therefore, if the valve can be switched on and off, the analysis time can be shortened. We therefore tested whether the valve could be opened and closed reversibly. A performance test was conducted using the valve with 1.48 μg/mm^2^ of polymerization. Two minutes after the colored water was injected through the import, the valve was heated. After 60 s from the start of heating, the membrane was switched to cooling and the change in the velocity of the colored water flowing through the upper channel was examined. A video of this process is shown in Video S4. The distance traveled by the wetting front of green colored water in the upper channel was measured every 10 to 30 s (Figure 10a), and flow velocity was calculated from the difference between distance traveled and the distance traveled at the previous measurement point (Figure 10b). The flow velocity was extremely low between 20 and 30 s after the beginning of cooling. The valve should have closed, but the flow velocity did not reach 0 mm/min because the liquid remaining in the upper channel flowed by capillary force. However, this flow velocity is considered to have little effect on sample analysis. It was also found that the valve opens again between 10 and 20 s after heating, and the liquid begins to flow. The valve responded to repeated cycles of cooling and heating. In the third cooling cycle, the decrease in flow velocity was slower than in the first and second cooling cycles. This was probably because the amount of liquid remaining in the upper channel after the valve was closed increased, so that the dry part of the upper channel was able to suck up more liquid even after the valve was closed. The flow velocity after heating slowed down with each cycle. The flow velocity through the porous membrane is determined by Washburn’s Equation as

(1) $$L^{2}=\gamma Dt/4\mu$$

where *L* is distance traveled of the wetting front, *γ* is the effective surface tension, which includes the effect of any contact angle dependence, *D* is the average pore diameter, and *µ* is viscosity [27,28,29]. Differentiating this shows that the flow velocity is inversely proportional to the square root of time. Focusing on the flow velocity change during each heating time, the flow velocity increased with the time for the second and third heating, contrary to Washburn’s Equation. It is considered that this is due to the pores in the valve having been gradually opening as the heating time elapsed. On the other hand, during the first heating, the flow velocity after 30 s was faster than that after 60 s. This is because the origin when calculating the travel distance is set at the lower edge of the upper channel, and immediately after the first heating, the overlapping area of about 5 mm width between the upper and lower channels was wetted at once, resulting in a larger travel distance.

## 4. Conclusions

In this study, thermo-responsive valves were fabricated by plasma-induced graft polymerization of PNIPAAm thermo-responsive resin onto PVDF porous membranes to control flow on a paper channel. The performance of the proposed valves was evaluated in detail for different amounts of membrane polymerization. We confirmed that the fabricated PVDF-PNIPAAm membranes worked well as valves when the amount of polymerization in the membrane was between 0.61 and 1.48 μg/mm^2^. In addition, these valves allow for protein permeability, suggesting that they are applicable to ELISA. Furthermore, it was shown that the proposed valves can be reversibly opened and closed. These results indicate that the proposed valves could be applied to the automation of ELISA with μPADs, and contribute to increasing the accuracy and shortening the analysis time of these types of testing devices. We plan to perform ELISA using this valve to demonstrate the usefulness of this valve.

## Data Availability

All the data generated or analyzed during this study are included in this published article.

## Supplementary Materials

The following supporting information may be downloaded at: https://www.mdpi.com/article/10.3390/mi13050690/s1, Video S1: Valve performance test of the valve with 0.1 μg/mm^2^ of polymerization, Video S2: Valve performance test of the valve with 0.97 μg/mm^2^ of polymerization, Video S3: Valve performance test of the valve with 2.85 μg/mm^2^ of polymerization, Video S4: Valve reversibility test of the valve with 1.48 μg/mm^2^ of polymerization.
